# Sex differences in the association of emotional approach coping with stress and quality of life among patients with renal cell carcinoma

**DOI:** 10.1017/S1478951525101569

**Published:** 2026-01-30

**Authors:** Chelsea G. Ratcliff, Robin Semelsberger, Surena F. Matin, Nizar M Tannir, Eric Jonasch, Louis L. Pisters, Lorenzo Cohen

**Affiliations:** 1 Department of Psychology, University of Houston, Houston, TX, USA; 2 Department of Psychology & Philosophy, Sam Houston State University, Huntsville, TX, USA; 3 Department of Urology, Division of Surgery, The University of Texas MD Anderson Cancer Center, Houston, TX, USA; 4 Department of Genitourinary Medical Oncology, Division of Cancer Medicine, The University of Texas MD Anderson Cancer Center, Houston, TX, USA; 5 Department of Palliative, Rehabilitation and Integrative Medicine, Division of Cancer Medicine, The University of Texas MD Anderson Cancer Center, Houston, TX, USA

**Keywords:** Renal cell carcinoma, emotional-approach coping, emotional expression, perceived stress, quality of life, sex

## Abstract

**Objectives:**

Emotional-approach coping (EAC), including emotional expression (EE) and emotional processing (EP), may impact stress and quality of life (QOL) in cancer populations, with some evidence that EAC effects vary by sex.

**Methods:**

Men (*n* = 85) and women (*n* = 63) with renal cell carcinoma (RCC) completed the EAC Scale, Perceived Stress Scale (PSS), and 36-item Medical Outcomes Study Short Form Survey (SF-36) physical component scale (PCS) and mental component scale (MCS) at study entry and 10 months later. The PROCESS macro (model 7) was used to examine the indirect effect of baseline EAC (EE, EP) on 10-month QOL (PCS, MCS) via baseline PSS, with sex as a moderator of the association between EAC and PSS (i.e., four models of moderated mediation).

**Results:**

Bootstrap estimates of indirect effects revealed significant moderated mediation, such that, for female participants, greater EE at study entry was associated with lower PSS, which in turn was associated with higher PCS and MCS 10 months later; whereas for males, EE was not associated with PSS and was not indirectly associated with physical and mental health-related QOL via PSS. Models examining the indirect effects of EP on QOL via PSS were nonsignificant for male and female participants.

**Significance of results:**

EE is an important correlate of perceived stress for females but not males with RCC. Perceived stress early in treatment has a robust association with subsequent health-related QOL. Interventions aimed at supporting EE for females with RCC may have long-term QOL benefits.

## Introduction

Cancer diagnosis, treatment, and the transition to survivorship are chronic stressors that can increase risk for compromised quality of life (QOL). Treatment for renal cell carcinoma (RCC) has seen particular improvement in recent years, which is associated with increased longevity but also introduces new treatment- and survivorship-related burdens that impact health-related QOL (St-Laurent et al. 2025). For example, one study found that nearly all participants with kidney cancer (92%) reported moderate or high perceived stress (Demirtaş and Temircan 2021) and a meta-analysis reported depression and anxiety symptoms among 78% and 68% of patients with non-metastatic RCC, respectively, with many studies noting up to half of patients reported moderate or severe mood symptoms (Vartolomei et al. 2022). However, relatively little research has examined the impact of various coping approaches on distress and QOL among patients with RCC. Perceived stress is associated with compromised mental and physical health-related QOL (Antoni and Dhabhar 2019; Dehghan et al. 2020). Additionally, among patients with more advanced disease, it is also associated with increased risk of progression, recurrence, and mortality (Bergerot et al. 2019), likely due to the immunosuppressive effect of chronic psychological stress (Moreno-Smith et al. 2010). In fact, depression has been found to increase risk of mortality in RCC and was likely mediated by stress-related immune and inflammatory factors (Cohen et al. 2012). Complementary strategies to manage stress has been associated with improved immune function and better QOL among patients with cancer (Abrahão et al. 2019). Thus, identifying effective strategies to manage and reduce stress may lead to improved mental and physical health.

### Emotional approach coping

Coping strategies are techniques used by an individual to manage stress and psychological strain (Litman and Lunsford 2009). Lazarus and Folkman (1987) put forth a model of coping that classified coping strategies as problem-focused, aimed at directly addressing the stressor, or emotion-focused, aimed to manage the resulting emotional distress caused by the stressor. Studies have found that emotion-focused coping strategies were considered more adaptive for stressors that are unpredictable and cannot be planned for, such as health-related stress (Litman and Lunsford 2009; Janowski et al. 2014). Therefore, it may be especially beneficial to examine the impact of emotion-focused coping on mental and physical health among RCC patients.

Given that some emotion-focused coping strategies can be adaptive (e.g., acceptance) and some maladaptive (e.g., denial), Stanton and colleagues (Stanton et al. 2000b) proposed a new category of coping strategies with more precise parameters, which they called emotional-*approach* coping (EAC; Austenfeld and Stanton 2004). EAC is conceptualized as an adaptive tendency to cope with stressful experiences through emotional expression (EE) and emotional processing (EP; Austenfeld and Stanton 2004; Juth et al. 2015). EE is characterized by active attempts to communicate one’s emotional experience verbally and/or nonverbally whereas EP describes active effort to acknowledge, explore, and understand one’s emotional experiences. A recent meta-analysis of 86 studies of EAC reported a significant association of EAC with overall health (Hoyt et al. 2024), and many studies have illustrated its association with self-reported and physiologically assessed health (Master et al. 2009; Moreno et al. 2016), and including among individuals with cancer (Giese-Davis et al. 2006; Stanton et al. 2000a).

Interestingly, EE and EP often have different effects on outcomes (Stanton et al. 2000b; Hoyt et al. 2024) and these may depend on sex (Stanton and Low 2012b). For example, studies in women with breast cancer found EE was associated with less distress, fewer depressive symptoms, and improved life satisfaction, whereas EP predicted higher distress, increased depressive symptoms, and reduced life satisfaction in highly expressive women (Stanton et al. 2000a; Stanton and Low 2012a). Similarly, another study found that in women with medical problems EP was positively associated with rumination and pervasive worry (Segerstrom et al. 2003). However, another study of women with gynecologic cancer found that both EE and EP were associated with lower depression, but EE was also associated with higher cortisol (Siwik et al. 2020). Conversely, in men with prostate cancer, EP predicted lower levels of inflammation and increased immunocompetence (Hoyt et al. 2013a) and declines in EP were associated with poorer prostate-specific physical functioning (Hoyt et al. 2013b). However, another study found that EP was associated with more distress among young men with cancer (Hoyt 2009). Thus, the impact of EE and EP in sex-specific cancer populations is somewhat equivocal.

Several studies have directly examined sex differences in the effects of EAC. For example, low EE and EP was associated with greater distress for females than males in a sample of couples experiencing infertility (Berghuis and Stanton 2002). Similarly, in a study of chronic pain patients, EE was associated with reduced pain interference and less negative affect for female but not male participants (Ziadni et al. 2020). Another sample of college undergraduates similarly found that EP and EE were associated with higher life satisfactions for females but not for males (Stanton et al. 2000b). Contrary to these findings, a study of cancer survivors found that EE was associated with lower intrusive thoughts for males, whereas EP was associated with higher positive affect for female survivors (Cho et al. 2013). Taken together, EE and EP have demonstrated differences which may vary by sex. Researchers have purported that health outcomes associated with EP can be positive or negative depending on its purposefulness, whereas EE has shown consistently positive health outcomes, particularly for females.

### The present study

The present study was a secondary analysis of a randomized clinical trial conducted at the MD Anderson Cancer Center examining the impact of an expressive writing intervention, in which patients were instructed to write about their “deepest thoughts and feelings related to their cancer” for 20 minutes on four separate occasions over a 10-day period, compared to a neutral writing (NW) condition, in which patients were instructed to write about dietary behaviors, physical activity and exercise behaviors, attitudes toward smoking and other substance use, and sleep habits on health outcomes and QOL in patients with RCC (Milbury et al. 2014, 2017). Previously published findings from this dataset demonstrated that patients participating in the expressive writing intervention reported lower cancer symptoms and higher physical health-related QOL 10-month post-intervention compared to the NW control group.

Currently no research has examined the effects of EAC (EE, EP) in patients with RCC. The primary aim of this study was to examine the indirect association of EAC (EE, EP) at study entry with health-related QOL assessed 10 months later via reduced perceived stress in patients diagnosed with RCC. Sex differences were explored as a moderator of the indirect and direct effects (i.e., moderated mediation). Specifically, we examined sex as a moderator of the association of EAC with perceived stress and with health-related QOL in the context of the mediation model. It was hypothesized that EE and EP would be associated with lower PSS, which in turn would be associated with higher PCS and MCS. Additionally, the present study examined sex as a moderator of this indirect effect. In particular, it was hypothesized that the indirect effect of emotional approach coping on subsequent health-related QOL via perceived stress would be greater for females than for males.

## Method

### Participants

Researchers approached 761 adult patients recently diagnosed with RCC stage I–IV, 355 consented to participate in the study either 6-month post-surgery or at initial consult prior to treatment, 284 participants completed the baseline questionnaires relevant to this study (EAC, PSS, and SF-36) and 148 participants completed the 10-month follow-up questionnaires relevant to this study (SF-36 PCS and MCS). Only participants who provided data at baseline and 10 months were include (*N* = 148).

### Procedures

A full description of the study procedures can be found in the primary paper (Milbury et al. 2014). Following baseline, participants were assigned to an expressive writing condition or NW control condition. After completing the 10-day intervention, participants provided self-report questionnaires and blood samples 1-month, 4-month, and 10-month post-intervention. The current study examined the indirect association of baseline Emotional Approach Coping strategies of EE and EP with physical and mental health-related QOL (SF-36 PCS and MCS) assessed 10-months later via baseline perceived stress (PSS) and whether sex moderated the associations.

### Measures

*Demographic and Clinical Information.* Participants completed a demographic form as part of the survey including age, sex, ethnicity, race, income and education. Clinical characteristics including stage of the disease (I–IV), treatment type (surgery or systematic), and cell type (clear cell) were collected from patients’ medical records.

*Emotional-Approach Coping Scale* (EACS) is an 8-item scale assessing EAC strategies using two subscales: EE and EP (Stanton et al. 2000b). Participants are asked to “indicate what you generally do, feel, and think when you experience stressful situations.” The EE subscale reflects an individual’s attempts to cope through EE and communicate their emotional experiences, sometimes considered interpersonal EAC (e.g., “I take time to express my emotions”). The EP subscale reflects an individual’s attempts to cope through EP, as acknowledging, exploring, and trying to understand one’s emotions, sometimes considered intrapersonal EAC (e.g., “I take time to figure out what I’m feeling”). Responses are scored on a 4-point Likert scale from 1 (“I usually don’t do this at all”) to 4 (“I usually do this a lot”). The original construction and validity studies showed high internal consistency for both subscales: EE Cronbach’s alpha ranged .72 to .92 and EP Cronbach’s alpha ranged .72 to .91.

The *Perceived Stress Scale* (PSS-14) consists of 14 statements asking individuals how often they have perceived stress in the past month (e.g., “In the past month, how often have you felt nervous and stressed?”), Items are measured on a 4-point Likert scale from 0 (never) to 4 (very often). Validation studies have showed high internal consistency (Cronbach’s alpha at .84 and above) (Cohen et al. 1983).

*Medical Outcome Survey-Short Form* (SF-36) measures self-reported health-related QOL across eight domains: general health perceptions, vitality, physical health functioning, bodily pain, role limitations resulting from physical health problems, social functioning, mental health, and role limitations resulting from emotional problems. The RAND scoring method was used (0–100) with higher scores indicating better health states, and the physical component summary (PCS) and mental component summary (MCS) scores were calculated. The SF-36 has previously produced good internal consistency (Cronbach’s alpha for various subscales ranging .78–.93) and good test-retest reliability (correlation coefficient ranging .60–.81) (Ware et al. 2000).

### Data analyses

Prior to inferential analyses, descriptive characteristics (e.g., frequency distributions, ranges, means, standard deviations, measures of skewness and kurtosis) of the baseline and 10-month data was reviewed to confirm assumptions for inferential statistics were met. Bivariate associations between demographic/clinical variables and the outcome variables (PCS and MCS) were conducted to determine covariates. *T*-tests were conducted to determine sex differences on study variables to further characterize the sample. All study variables were then standardized to ease interpretation of the mediation analyses. All models described below covaried for group assignment (EW vs. NW), the baseline level of the outcome variable (PCS or MCS), and clinical or demographic characteristics found to be significantly associated with study variables.

To evaluate all the hypotheses for the proposed study, four moderated mediation models were conducted using the PROCESS Macro for SPSS (Model 8) (Rucker et al. 2011; Hayes 2013). Using the conditional indirect mediation model (Model 8), a 5000-sample bootstrap procedure was be used test the indirect effects of each predictor variable (baseline EE and EP) on each of the dependent variables (10-month PCS and MCS) via the mediator (baseline PSS), with sex included as a moderator of the association of EAC with PSS (i.e., the “a” path) and with PCS and MCS (i.e., the “c” path). Direct and indirect effects and the index of moderated mediation were determined significant when the mean of the indirect effect across all 5000 bootstrap samples was associated with a bias-corrected confidence interval that did not include 0.

Power was estimated using the method proposed by Pan et al. (2018) to determine the sample size needed to detect mediation effects using longitudinal data. Given moderate interclass correlations between measures at the two time points (ICC of .6), a sample size of 130 would allow a simple indirect effect to be detected as statistically significant at alpha of .05 with 80% power using a bootstrapped approach if the associations between the predictor and mediator and between the mediator and outcome were small to moderate (e.g., *b* = .26).

## Results

### Sample characteristics

Participant characteristics can be seen in Table 1 and means of and correlations among study variables can be seen in Table 2. Only participants who provided data at baseline and 10-months were included (*N* = 148). Most participants were White/European American (78%), partnered (77%), employed (63%), with clear cell type (81%), and surgery as the treatment (70%). Most were within 6 months of diagnosis (79%), with over half being within 3 months of diagnosis (55%). Close to half participants earned more than $75,000 per year (49%), attended college (49%), were male (57%), and had Stage 1 disease (43%). Participants mean age was 57.9 years (SD ± 9.9 years, range 31–82 years). There were no systematic differences between those with complete data (*N* = 148) and those without (*N* = 129) on any demographic, clinical, or self-report data, with two exceptions: completers were less likely to have advanced stage (22% of completers had versus 40% of non-completers; *p* = .008) and less likely to have systemic treatment (30% of completers had systemic treatment versus 46% of non-completers; *p* = .006).

**Table 1.** Sample characteristics

	*N* = 148	%
**Sex**		
Male	85	57.4
Female	63	42.6
**Race/ethnicity**		
White	116	78.4
Hispanic	18	12.2
Other	11	7.4
Missing	3	2
**Marital status**		
Partnered	114	77
Unpartnered	30	20.3
Missing	4	2.7
**Employment status**		
Employed	93	62.8
Unemployed	52	35.1
Missing	3	2
**Income**		
<$75,000	68	45.9
≥$75,000	72	48.6
Missing	8	5.4
**Education**		
College	73	49.3
No college	71	48
Missing	4	2.7
**Stage**		
1	63	42.6
2	22	14.9
3	27	18.2
4	32	21.6
Missing	4	2.7
**Treatment type**		
Surgery	104	70.3
Systemic treatment	44	29.7
**Cell type**		
Clear cell	120	81.1
Other	26	17.6
Missing	2	1.4
**Time since diagnosis**		
<3 months	81	54.73
3–6 months	36	24.32
>6 months	24	16.22
Missing	7	4.73

**Table 2.** Means and correlations between study variables

				Pearson correlation Coefficients						
	Variable	M	SD	1	2	3	4	5	6	7
Baseline	1. EE	11.61	2.56	1						
	2. EP	11.10	3.01	.527**	1					
	3. PSS	18.10	8.15	0.042	−0.139	1				
	4. PCS	39.78	10.40	−.176*	−.189*	−0.148	1			
	5. MCS	48.38	9.05	−.173*	0.096	−.617**	−.259**	1		
10 months	6. PCS	44.18	11.99	−0.133	−0.112	−.230**	.488**	−0.048	1	
	7. MCS	48.53	7.86	−0.089	.177*	−.421**	−0.03	.435**	−.172*	1

Note: EE = Emotional Expression, EP = Emotional Processing, PSS = Perceived Stress Scale, PCS = SF-36 Physical Component Scale, MCS = SF-36 Mental Component Scale;

** Correlation is significant at the 0.01 level (2-tailed). * Correlation is significant at the 0.05 level (2-tailed).

### Determining covariates

Physical health-related QOL (PCS) at 10 months was higher among individual who were younger (*r* = − .24, *p* = .004), employed versus not employed (47.5 vs. 38.7, *p* < .001), had attended college versus not (47.3 vs. 41.1, *p* = .001), had income over versus under $75,000 (47.3 vs. 41.3, *p* = .003), stage 1–3 vs. stage 4 disease (45.2 vs. 38.7, *p* = .012), surgery versus systemic treatment (46.9 vs. 37.8, *p* = < .001), surgery type (partial vs. full nephrectomy) (45.6 vs. 39.2, *p* = .007), and were assigned to the expressive writing versus NW condition (46.2 vs. 42.3, *p* = .045). Thus, when PCS was the outcome variable, covariates included age, employment, education, income, stage, treatment, surgery, study condition, and baseline PCS.

Mental health-related QOL (MCS) at 10 month was higher among individuals with income under versus over $75,000 (49.7 vs. 47.3, *p* = .047). No other clinical or demographic variables were associated with 10-month MCS. Thus, when MCS was the outcome variable, covariates included income, study condition (to ensure accounting for effect of intervention) and baseline MCS.

### Sex differences in emotional approach coping, perceived stress, and QOL

Sex differences in study variables can be seen in Table 3. Females reported higher baseline EE (*p* > .001) and EP (*p* = .029) compared to males, and lower baseline PCS (*p* = .036). Male and female participants reported similar baseline perceived stress, baseline MCS, and 10-month PCS and MCS.

**Table 3.** Sex differences in study variables

		Female (*n* = 63)		Male (*n* = 85)		
		*M*	SD	*M*	SD	*p*-Value*
Baseline	EE	12.08	2.95	10.39	2.85	<.001
	EP	12.15	2.57	11.21	2.50	0.029
	PSS	18.87	9.28	17.54	7.22	0.331
	PCS	37.65	9.81	41.31	10.60	0.036
	MCS	49.09	8.69	47.86	9.31	0.418
10 months	PCS	42.18	12.86	45.67	11.14	0.080
	MCS	48.85	8.74	48.29	7.19	0.666

Note: EE = Emotional Expression, EP = Emotional Processing, PSS = Perceived Stress Scale, PCS = SF-36 Physical Component Scale, MCS = SF-36 Mental Component Scale;

* *p*-value from independent *t*-tests.

### Indirect association of emotional approach coping with hrqol via perceived stress: Sex effects

**Figure 1.**
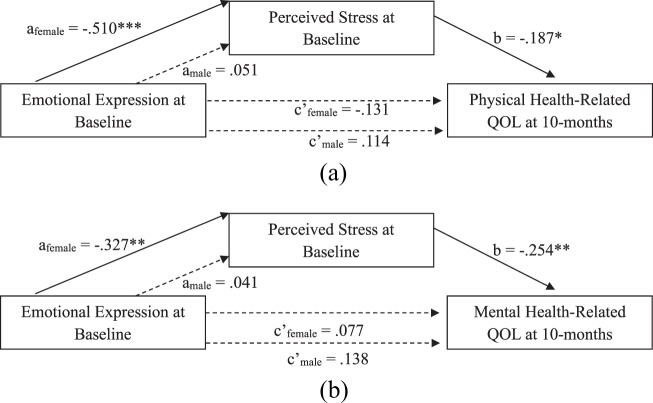
The indirect effect of emotional expression on HRQoL via perceived stress depends on sex. (a) *Note*: Dashed line indicates non-significant path; solid line indicates significant path. Model covaries for age, employment, education, income, stage, treatment, surgery, study condition, and baseline PCS. *p < .05, ***p < .001. (b) *Note*: Dashed line indicates non-significant path; solid line indicates significant path. Model covaries for income, study condition, and baseline MCS. *p < .05, **p < .01.

**Figure 2.**
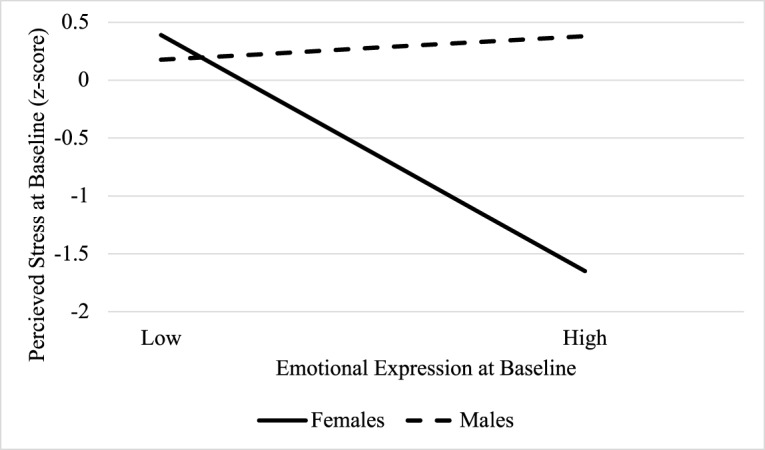
Association of emotional expression with perceived stress depends on sex.

PROCESS model 8 revealed significant moderated mediation, such that the indirect association of baseline EE on 10-month PCS via baseline PSS depended on sex (index of moderated mediation: −.10, 95% CI [−.23, − .01]; Fig. 1a). Specifically, the association of baseline EE on PSS was conditional on sex (*β* = .56, 95% CI [.18, .95], *p* = .005), such that baseline EE was negatively associated with PSS for *f* (*β* = − .51, 95% CI [−.81, −.21], *p* < .001), but not men (*β* = .05, 95% CI [−.20, .30], *p* = .69) (Fig. 2). PSS was, in turn, negatively associated with 10-month PCS (*β* = − .19, 95% CI [−.33, − .05], *p* = .010). The association of EE with PCS did not depend on sex (*p* = .13); there was no evidence for a direct effect of EE on PCS for females (*β* = − .13, 95% CI [−.38, .12], *p* = .29) or males (*β* = .11, 95% CI [−.09, .31], *p* = .26). However, examination of conditional indirect effects of baseline EE on 10-month PCS via PSS revealed significant mediation only for females (indirect effect: .10, 95% CI [.02, .20]). In other words, for female participants, higher EE was associated with lower PSS, which in turn was associated with lower PCS scores.

The indirect association of baseline EE on 10-month MCS via baseline PSS also depended on sex (index of moderated mediation: −.09, 95% CI [−.22, − .01]; Fig. 1b). As in the previous model, the association of baseline EE on PSS was conditional on sex (*p* = .017), such that baseline EE was negatively associated with PSS for females (*β* = − .33, 95% CI [−.56, − .10], *p* = .006), but not males (*β* = .04, 95% CI [−.15, .23], *p* = .67). PSS was, in turn, negatively associated with 10-month MCS (*β* = − .25, 95% CI [−.45, − .05], *p* = .014). The association of EE with MCS did not depend on sex (*p* = .74); there was no evidence for a direct effect of EE on PCS for females (*β* = .08, 95% CI [−.29, .35], *p* = .58) or males (*β* = .14, 95% CI [−.09, .36], *p* = .23). Further, examination of conditional indirect effects of baseline EE on 10-month MCS via PSS revealed significant mediation only for females (indirect effect: .08, 95% CI [.01, .18]). In other words, for female participants, higher EE was associated with lower PSS, which in turn was associated with lower MCS scores.

There was no evidence for a direct or indirect effects of EP on PCS or MCS. Specifically, there were no main effects of baseline EP or an EP by sex interaction effect on baseline PSS or 10-month PCS or MCS. Thus, an indirect effect of EP on health-related QOL via reduced PSS was not supported for females or males.

## Discussion

The present study’s results suggest an indirect effect of EE on subsequent physical and mental health-related QOL via lower perceived stress for female, but not males, with RCC (i.e., moderated mediation). Specifically, for females, greater endorsement of EE in response to the stressful experience of cancer treatment (e.g., “I let my feelings come out freely,” “I allow myself to express my emotions”) was associated with lower concurrently assessed perceived stress; however, EE was unrelated to perceived stress for males. This finding is consistent with several studies that suggest EE is especially beneficial for females compared to men in the general population (Stanton et al. 2000b), as well as in the context of medical stressors including infertility (Berghuis and Stanton 2002), chronic pain (Ziadni et al. 2020), and cancer (Stanton et al. 2000a; Stanton and Low 2012a). Some researchers have posited that sex-related social constraints may explain sex-differences in the impact of EE. Specifically, it may be that EE from females is perceived as more consistent with sex expectations and therefore more well received than when males use EE as a coping strategy (Hoyt 2009). In this study, females did report greater use of EE and EP as ways to cope with stress compared to males, though males still reported in engaging in moderate amounts of both forms of emotional approach coping (EAC). This is consistent with studies that have found that females use more EAC compared to males in clinical (Dev et al. 2024) and nonclinical samples (Graves et al. 2021). Further, several studies have found that interventions designed to facilitate EAC are particularly beneficial for individual already prone to coping with stress via EAC (Niles et al. 2014; Seeley et al. 2017). Thus, it may be that EE was more consistent with the (possibly socialized) coping preferences of females in this study and therefore a stronger correlate of stress for females.

Importantly, the present study did not find that EE at study entry directly affected 10-month follow-up QOL for males or females in the absence of perceived stress, which suggests that the impact of EE on female’s acute experience of stress is an important consideration. Indeed, previous research suggests that EE in response to a stressful situation is associated with reduced self-reported and physiologically assessed stress. For example, affect labeling led to decreases in self-reported distress and limbic activity in response to experimentally induced stress (Burklund et al. 2014). Additionally, expressive writing interventions, which explicitly aim to increase EE, have been shown to improve within-session heart rate variability (Seeley et al. 2017) and heart rate habituation (Low et al. 2008). Indeed, heart rate habituation during expressive writing sessions (Low et al. 2006) and reductions in perceived stress following expressive writing sessions (Lu et al. 2023) have both been identified as mechanisms of the interventions’ effect on subsequent mental and physical health symptoms in two studies of breast cancer survivors. Thus, this study’s results echo previous findings and suggests that EE in response to a stressor appears to be associated with lower stress, a robust predictor of long-term physical and mental health (Mazor et al. 2019; Dehghan et al. 2020).

The present study did not find EP to be associated with concurrent stress or 10-month QOL for males or females. Previous research on sex-differences in the impact of EP is a bit more equivocal, with some studies suggesting EP may be associated with positive affect for female cancer survivors (Cho et al. 2013) and better immune functioning among males with prostate cancer (Hoyt et al. 2013a) (Hoyt et al. 2013b), whereas other studies suggest EP may be correlated with more distress and rumination among females (Segerstrom et al. 2003) (Stanton et al. 2000a; Stanton and Low 2012a) and males with cancer (Hoyt 2009). Additional research remains necessary to identified factors that may influence the impact of EP among cancer survivors.

This study has several important limitations. First, the sample was predominantly White, which may limit generalizability of findings, particularly given evidence the impact of expressing emotions to close others depends on culture (Taylor et al. 2007). Second, this is a secondary analysis of an expressive writing intervention, which may have shaped the initially recruited sample as well as the sample retained at 10 months. Thus, the results may not generalize to RCC patients disinclined to participate in an expressive writing intervention. Third, the study did not experimentally manipulate EAC and, though the outcome (health-related QOL) was assessed 10-month post-study entry, EAC was assessed concurrently with the examined mediator (perceived stress). Thus, this study design is correlational, and results cannot determine causal associations between study variables.

## Conclusions

Overall, results suggest that engaging in EE in response to a stressor is associated with lower stress for females, but not males, with RCC. Lower stress, in turn, was an important correlate of subsequent mental and physical health-related QOL. Taken together, this study suggests that greater efforts to freely express emotions in the face of stress may be associated with improved long-term QOL via reduced stress for females with RCC. Interventions aimed at supporting EE for females with RCC may have long-term QOL benefits.
